# Fibroblast Growth Factor 21 Relieves Lipopolysaccharide-Induced Acute Lung Injury by Suppressing JAK2/STAT3 Signaling Pathway

**DOI:** 10.1007/s10753-023-01905-3

**Published:** 2023-10-21

**Authors:** Mengsi Cai, Huihui Ye, Xiayan Zhu, Xiuchun Li, Luqiong Cai, Jiajia Jin, Qiwen Chen, Yuzhe Shi, Lehe Yang, Liangxing Wang, Xiaoying Huang

**Affiliations:** 1 Division of Pulmonary Medicine, the First Affiliated Hospital, Wenzhou Medical University, Wenzhou Key Laboratory Interdiscipline and Translational Medicine, Wenzhou Key Laboratory of Heart and Lung, Wenzhou Medical University, Xuefu North Street, Wenzhou, Zhejiang 325000 People’s Republic of China; 2 The First Clinical Medical College, Wenzhou Medical University, Wenzhou, Zhejiang 325000 People’s Republic of China

**Keywords:** Acute lung injury (ALI)/acute respiratory distress syndrome (ARDS), FGF21, JAK2/STAT3 signal pathway.

## Abstract

Acute lung injury (ALI)/acute respiratory distress syndrome (ARDS) is a life‐threatening disease without an effective drug at present. Fibroblast growth factor 21 (FGF21) was reported to be protective against inflammation in metabolic disease in recent studies. However, the role of FGF21 in ALI has been rarely investigated. In this study, it was found that the expression of FGF21 was markedly increased in lung tissue under lipopolysaccharide (LPS) stimulation *in vivo*, whereas it was decreased in lung epithelial cells under LPS stimulation *in vitro*. Therefore, our research aimed to elucidate the potential role of FGF21 in LPS-induced ALI and to detect possible underlying mechanisms. The results revealed that the deficiency of FGF21 aggravated pathological damage, inflammatory infiltration, and pulmonary function in LPS-induced ALI, while exogenous administration of FGF21 improved these manifestations. Moreover, through RNA sequencing and enrichment analysis, it was unveiled that FGF21 might play a protective role in LPS-induced ALI via JAK2/STAT3 signaling pathway. The therapeutic effect of FGF21 was weakened after additional usage of JAK2 activator *in vivo*. Further investigation revealed that FGF21 significantly inhibited STAT3 phosphorylation and impaired the nuclear translocation of STAT3 *in vitro*. In addition, the aggravation of inflammation caused by silencing FGF21 can be alleviated by JAK2 inhibitor *in vitro*. Collectively, these findings unveil a potent protective effect of FGF21 against LPS-induced ALI by inhibiting the JAK2/STAT3 pathway, implying that FGF21 might be a novel and effective therapy for ALI.

**Supplementary Information:**

The online version contains supplementary material available at 10.1007/s10753-023-01905-3.

## INTRODUCTION

Acute lung injury (ALI)/acute respiratory distress syndrome (ARDS) is a critical respiratory disease characterized by progressive respiratory distress and intractable hypoxemia, which is caused by various direct or indirect injuries. The mortality rate of ARDS has remained at approximately 40% over the past 20 years [1]. Moreover, ALI/ARDS is one of the most serious complications of novel coronavirus pneumonia [2]. Although treatment strategies to alleviate ALI/ARDS have greatly improved in recent times, there is still a lack of efficient curative treatments.

Elevated inflammatory response is an important pathophysiological process in ALI. Various stimuli can induce neutrophil activation, promote inflammatory cell proliferation, and produce a large number of inflammatory factors [3]. Among these, lipopolysaccharide (LPS), found in the outer membrane of Gram-negative bacteria, has been identified as an important factor in the development of ALI/ARDS [4]. Therefore, animal model of LPS-induced ALI can be used as a clinically relevant model.

Fibroblast growth factor 21 (FGF21), an endocrine member of the fibroblast growth factor family [5], is predominantly expressed in the liver, pancreas, and adipose tissue [6–8]. Previous studies on FGF21 have focused on its role in various metabolic diseases [9–11]. It was found that circulating levels of FGF21 were increased in patients with sepsis and systemic inflammatory response syndrome (SIRS), which suggested a possible role of FGF21 in inflammation [12]. Moreover, Yu *et al*. found that FGF21 inhibited macrophage-mediated inflammation by activating Nrf2 and suppressing the NF-κB signaling pathway [13]. In addition, a previous study showed that FGF21 ameliorated LPS-induced dysfunction and inflammatory response of primary human pulmonary microvascular endothelial cells (HPMECs) through SIRT1-mediated NF-κB deacetylation [14]. Thus, while FGF21 appears to play a significant role in LPS-induced ALI, the exact mechanism remains to be elucidated.

Several studies have shown that cytokine release syndrome is an important mechanism underlying ALI/ARDS [15, 16]. Similarly, the result of bioinformatics analysis in our study indicated that cytokine-cytokine receptor response and the JAK2/STAT3 pathway were highly relevant to the function of FGF21 in mice with LPS-induced ALI. Interleukin-6(IL-6) plays a key role in cytokine release syndrome and is reported to serve as an indicator of cytokine storm severity and prognosis [17]. Binding of IL-6 to its cell membrane receptor can directly activate the JAK2/STAT3 pathway, which in turn promotes the expression of a variety of genes associated with proliferation, differentiation, apoptosis, and immune responses [18–20]. Ying *et al*. ﻿found that sodium butyrate relieved lung ischemia–reperfusion injury by inhibiting the NF-κB and JAK2/STAT3 signaling pathways [21]. Thus, based on the results of the literature survey and bioinformatics analysis, we hypothesized that FGF21 might relieve LPS-induced ALI by suppressing JAK2/STAT3 signaling pathway.

In this study, the differential expression of FGF21 after stimulation by LPS attracted our interest. We sought to comprehensively investigate the specific role of FGF21 in LPS-induced ALI and to figure out the possible underlying mechanisms.

## METHODS

### Reagents

Anti-myeloperoxidase (MPO) antibody (Abcam, ab208670), p-STAT3 (Abcam, ab32143), STAT3 (Abcam, ab119352), p-JAK2 (Abcam, ab32101), JAK2 (Abcam, ab108596), p-STAT4 (CST, Tyr701) and STAT4 (CST, C46B10), and β-actin (Abcam, ab6276). The primers used in this study were purchased from Ruibo (Guangzhou, China). Fedratinib (Sellleck, TG101348). Coumermycin A1 (MCE, HY-N7452).

### Mice

Male wild-type (WT) C57BL/6 J mice (6–8 weeks, body weight 20–25 g) were purchased from Shanghai Slack Laboratory Animal Co, Ltd (Shanghai, China) and C57BL/6 J background FGF21 knockout (FGF21KO) mice (6–8 weeks, body weight 20–25 g) were obtained as a gift by Dr. Steve Kliewer (University of Texas Southwestern Medical Center, Dallas, TX, USA). FGF21KO mice were generated as described in previous research [22]. As shown in Supplementary Fig. 1 in Supplementary file 1, agarose gel electrophoresis was performed to identify FGF21^+/+^ mice, FGF21^+/−^ mice, and FGF21^−/−^ mice.

All mice were housed under standard conditions (temperature 20–24℃; 12-h light–dark cycle) and fed standard rodent diet and water in specific pathogen-free environment. All the animals care and experimental procedures have been approved by the Experimental Animal Ethics Committee of Wenzhou Medical University.

### ALI Mice Models and Treatment

Mice were randomly selected and held the mouth of the anesthetized animal under cover with anesthetic mask full of soaked cotton ball of diethyl (Tianjin Kemiou Chemical Reagent Co, Ltd.60297) for about 1 min. Then, the mice were given LPS or saline into the trachea within 1 min. All procedures related with diethyl ether were performed in a well-vented fume hood. WT mice and FGF21KO mice received intratracheal injection of LPS (Sigma, St. Louis, MO, USA) 10 mg/kg of body weight or same amount of saline to build ALI mice model or control. Then, mice were sacrificed after 24-h stimulation of LPS.

WT mice were randomly divided into 4 groups, including control group, LPS group, LPS + FGF21 (1 mg/kg) group, and LPS + FGF21 (3 mg/kg) group. FGF21, obtained from Prospec (CYT‐281, Prospec, Rehovot, Israel), was given intraperitoneally at dose of 1 mg/kg in LPS + FGF21 (1 mg/kg) group and 3 mg/kg in LPS + FGF21 (3 mg/kg) group 0.5 h after the intratracheal injection of LPS. The mice in control group and LPS group were given intraperitoneally saline instead 0.5 h after the intratracheal injection of saline or LPS. After 24-h intervention of LPS, all mice were sacrificed.

WT mice were randomly divided into 4 groups as follows: control group, LPS group, LPS + FGF21, and LPS + FGF21 + CA (Coumermycin A1). FGF21 was given at dose of 3 mg/kg. CA was given intraperitoneally at dose of 200 μg/kg after FGF21 treatment. All mice were sacrificed after LPS stimulation for 24 h.

All the experiments *in vivo* were performed in biological and technical triplicates.

### Lung Wet/Dry (W/D) Ratio

The middle lobe of the right lung was cut and the wet weight was recorded after the surface water was sucked out. Subsequently, the samples were dried at 60℃ for 48 h to record dry weight. The W/D was calculated to evaluate pulmonary edema.

### Bronchoalveolar Lavage Fluid (BALF) Analysis

The left lung was lavaged for 3 times with 200 μL PBS while the right lung was ligated. The above procedure was repeated for 4 times. The collected BALF was centrifuged at 3000 rpm at 4℃ for 10 min. Then, the supernatant was used to detect the total protein concentration by a protein analysis kit (herculesbio-rad laboratory, CA, USA) while the cell pellet was re-suspended in 50 μL PBS followed by cell count using hematocytometer.

### Hematoxylin and Eosin (H&E) Staining and Immunohistochemistry Analysis

The right lower lobe of the lung was excised and fixed with 4% formalin. The lung tissues were embedded with paraffin, sliced to 5 μm sections, and stained with H&E. Mice lung histopathology images were acquired using a microscope. Lung injury was analyzed by 2 pathologists blinded to the level of induced injury. HE lung injury scores were calculated by light microscopic analysis of 4 parameters including alveolar septal thickness, interstitial edema, infiltration of inflammatory cells, and alveolar congestion/collapse. Each parameter was categorized into four grades: 0 = normal, 1 ≤ 25%, 2 = 25–50%, 3 = 50–75%, and 4 ≥ 75%, and the mean score of the four parameters was used to represent the overall lung injury [23].

The immunohistochemistry analysis was performed following the anti-myeloperoxidase (MPO) antibody staining protocol in the previous study [24]. The immunohistochemistry analysis of slides was taken by ordinary optical microscope. The positive staining cells were calculated using Image J 6.0 software. Positive staining of MPO was expressed as positive cells per mm^2^ (cells/mm^2^) [25, 26].

### Real-time (RT) PCR Analysis

Total RNA was isolated from lung and liver samples and from BEAS-2B cells using the RNeasy mini kit (Qiagen, USA) and reverse-transcribed into cDNA using iScript cDNA Synthesis Kit. Then, amplification of cDNA was carried out by a real-time PCR (RT-PCR) (CFX96 Real-Time System, Bio-Rad, USA). β-actin served as endogenous controls. The primer sequences for the experiment are listed in Table 1 in Supplementary file 1.

### Enzyme-linked Immunosorbent Assay (ELISA)

The protein levels of FGF21 in the cell culture medium, lung tissue homogenate, and serum of mice were measured using Mouse FGF21 ELISA Kit (Abcam, ab212160) and Human FGF21 ELISA Kit (Abcam ab125966) according to the manufacturer’s protocol [27, 28].

### Assessment of Pulmonary Mechanics

The evaluation of pulmonary function was done as described previously [29]. Briefly, after anesthesia, the mice were sacrificed and tracheostomized using an 18-gauge metal cannula and linked with controlled small ventilator, which was connected to a computer system (flexiVent, ScireqInc., Montreal, Canada). Firstly, the lungs were inflated 4 times to standardize lung volume history. To establish the baseline, regular ventilation data was collected. Then, the mice were nebulized with acetylcholine in 0.9% saline (Sigma-Aldrich) at increased concentrations (3.125, 6.25, 12.5, 25, 50 mg/mL, respectively). After receiving each dose of MCh and 0.9% saline, the measurements were delivered every 15 s, over 5-min duration. The obtained parameters were the resistance of the respiratory system (Rrs), elastance (Ers), lung compliance (Crs), tissue damping, and tissue elastance.

### RNA Sequencing and Bioinformatic Analysis

RNA isolation and sequencing were performed by RiboBio Co. Ltd. Concisely, RNA was extracted and purified following routine protocols and verified for quality and integrity. Only high-quality RNA samples (OD260/280 = 1.8 ~ 2.2, OD260/230 ≥ 2.0, RIN ≥ 8.0, 28S:18S ≥ 1.0, > 1 μg) were used to construct sequencing library. Subsequently, the transcriptome library was prepared following TruSeqTM RNA sample preparation kit from Illumina (San Diego, CA) using 1 μg of total RNA. Thereafter, the purified RNA was subjected to first‐strand and second‐strand cDNA synthesis, then adaptor ligation and low‐cycle PCR were used for enrichment. After quantified using TBS380, paired-end RNA-seq sequencing library was sequenced with the Illumina NovaSeq 6000 sequencer (2 × 150 bp read length). The raw paired-end reads were trimmed and quality controlled using fastp (https://github.com/OpenGene/fastp) with default parameters. Clean reads were separately aligned to reference genome with orientation mode by HISAT2 (http://ccb.jhu.edu/software/hisat2/index.shtml) software.

The “limma” package was used for standardization. The differentially expressed genes (DEGs) were screened out using “limma” package by the following selection criteria: *P* value < 0.05 and fold change (FC) > 2. Then, the Gene Ontology (GO) including biological process (BP), molecular function (MF), and cellular component (CC) enrichment analyses, as well as Kyoto Encyclopedia of Genes and Genomes (KEGG) pathway enrichment analyses of the DEGs, was conducted with “clusterprofiler” package in R software. The results were visualized by “GOplot” package.

### Western Blot

Lung and liver samples were homogenized with RIPA buffer (Promega, Madison, WI, USA) using a FastPrep‐24 5G Instrument (MP Biomedicals, CA, USA). Then, the supernatants were collected. Protein concentrations were measured by a Pierce BCA protein assay kit (Thermo Fisher Scientific Inc., Rockford, IL, USA). Then, equal amounts of protein samples were separated by 10% SDS-PAGE and then electro-transferred to polyvinylidene fluoride (PVDF) membrane. After blocking for 90 min with 5% nonfat dry milk at room temperature, the membranes were incubated overnight at 4 °C with specific primary antibodies. The next day, after incubating with secondary antibodies, the proteins were treated with ECL reagent (Pierce, WI, USA) for blot detection.

### Cell Culture and Transfection

BEAS-2B was purchased from American Type Culture Collection (ATCC, Manassas, VA, USA) and cultured in Dulbecco’s Modified Eagle’s Medium (DMEM, Sigma, St. Louis, MO, USA) containing 10% fetal bovine serum (FBS, Gibco, Thermo Fisher Scientific, Waltham, MA, USA) with 5% CO2 under humidity of 95% at 37 °C. Cells were cultured in 6-well plates and allowed to acclimate for 24 h before further treatment.

In order to downregulate or overexpress FGF21 in BEAS-2B, negative control siRNA (NC siRNA) and 3 small interference sequences of FGF21 (si-FGF21-1, si-FGF21-2, and si-FGF21-3) to knockdown of FGF21 were ordered from Guangzhou Ruibo Company. According to the manufacturer’s protocols, BEAS-2B cells were transfected with different sequences of siRNA-FGF21 and the knockdown efficiency was verified by RT-PCR. In addition, overexpression of FGF21 and STAT3 in BEAS-2B cells was carried out by transfected with plasmids of pcDNA-FGF21 (Guangzhou Ruibo Company) and pcDNA-STAT3 (Guangzhou Ruibo Company). RT-PCR and Western blot were performed to detect the expression of FGF21 and STAT3 in order to determine the transfection efficiency. Fedratinib (3.2 nM) was added in culture medium of adhered BEAS-2B cells in an additional group to inhibit the JAK/STAT3 signal pathway. Then, cells were exposed to LPS (0.5 µg/mL) for 24 h.

All experiments *in vitro* were performed in biological and technical triplicates.

### Immunofluorescent Staining

BEAS-2B cells were cultured in 6-well plates. After transfection treatment mentioned above, the cells were fixed by 4% paraformaldehyde for 20 min at 4 °C. The cells were then washed three times with PBS and incubated in 1% Triton X-100 for 15 min and 1% BSA for 1 h at room temperature. Cells were then incubated overnight at 4 ℃ on addition of anti-P-STAT3 primary antibody solution (1:300 in 3% BSA). After rewashed in PBS, the cells were allowed to react with PE-labeled secondary antibody (1:300 in 3% BSA) for 1 h in a dark room and counterstained 4,6-diamidino-2-phenylindole dihydro-chloride (DAPI) for 5 min. Images were captured under the confocal microscope.

### Statistical Analysis

GraphPad Prism 6.0 (GraphPad Software, CA, USA) was performed for the statistical analysis. All data were analyzed using the KS normality test for normality and presented as the mean ± SD. Comparisons between 2 groups were analyzed by Student’s *t*-test, and multiple comparisons were analyzed by one-way ANOVA. Additionally, two-way ANOVA was used in assessment of lung function. *P < 0.05 and **P < 0.01, as indicated.

## RESULTS

### The Expression of FGF21 in the Lung, Serum, Liver, and Lung Epithelial Cells upon LPS Stimulation

**Fig. 1:**
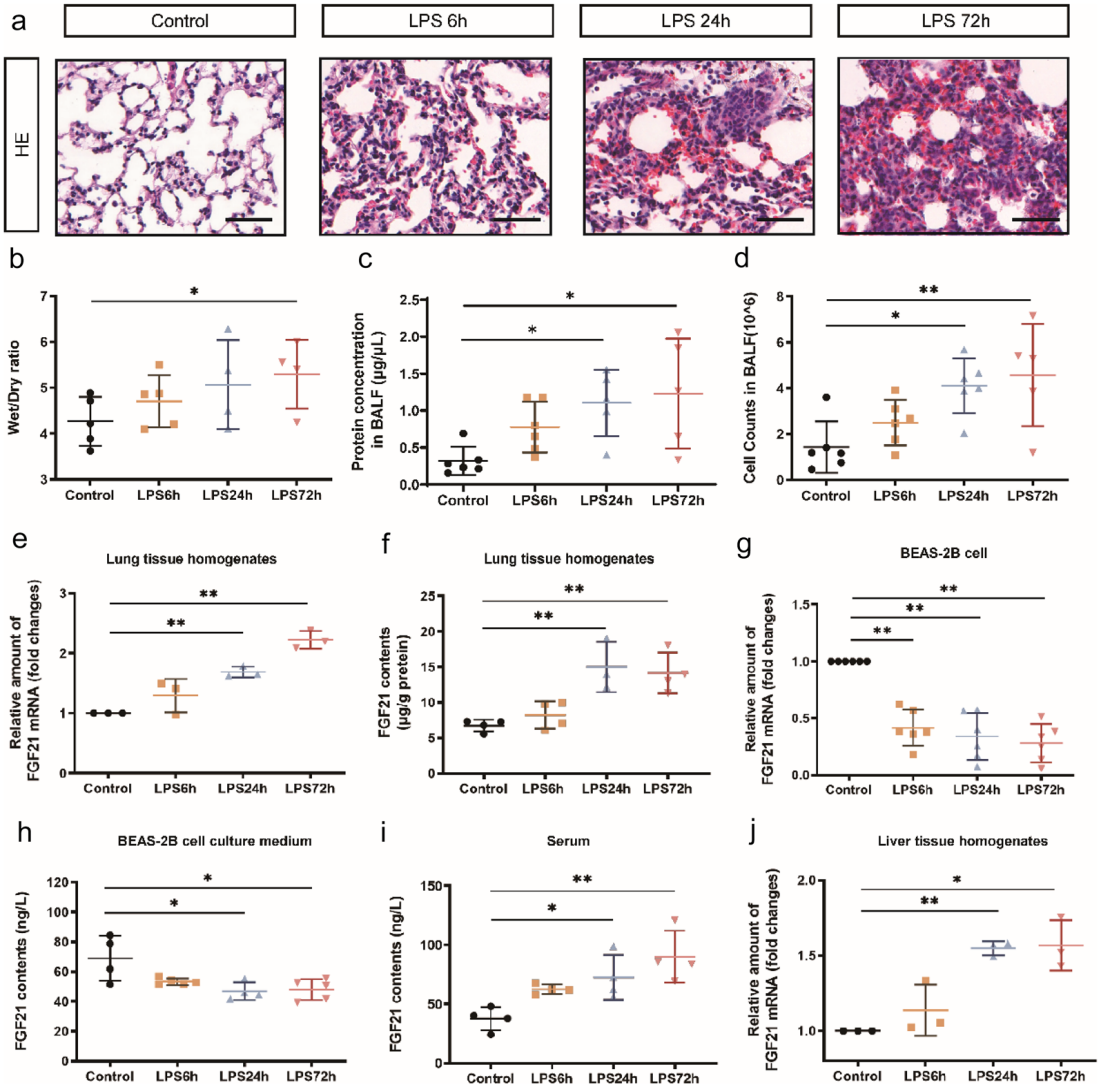
Opposite trend of FGF21 expression level in LPS-induced ALI model *in vivo* and *in vitro*. **a** Representative H&E staining image of the lung tissue of control group, LPS6h group, LPS24h group, and LPS72h group (*n* = 20), scale bar = 50 μm. **b** Lung wet/dry ratio of control group, LPS6h group, LPS24h group, and LPS72h group (*n* = 4–5). After bronchoalveolar lavage fluid (BALF) was collected, the levels of total secretory protein in BALF of control group, LPS6h group, LPS24h group, and LPS72h group were detected (*n* = 5–6) (**c**) and the cells in BALF of control group, LPS6h group, LPS24h group, and LPS72h group were counted (*n* = 5–6) (**d**). **e** Relative amount of FGF21 mRNA in lung tissue (10 mg) were detected by PCR of control group, LPS6h group, LPS24h group, and LPS72h group (*n* = 3). **f** FGF21 protein levels in lung tissue homogenate of control group, LPS6h group, LPS24h group, and LPS72h group detected by FGF21 Elisa kit (*n* = 3–4). **g** Relative amount of FGF21 mRNA in BEAS-2B cells challenged by LPS (0.5 µg/mL) for 6 h, 24 h, and 72 h (*n* = 6). **h** FGF21 in cell culture supernatant of control group, LPS6h group, LPS24h group, and LPS72h group were detected by FGF21 Elisa kit (*n* = 4–5). **i** FGF21 level in mice serum of control group, LPS6h group, LPS24h group, and LPS72h group were detected by kit (*n* = 3–4). **j** Relative amount of FGF21 mRNA in liver tissue (10 mg) of control group, LPS6h group, LPS24h group, and LPS72h group determined by PCR (*n* = 3). Multiple comparisons were analyzed by one-way ANOVA. *P < 0.05 and **P < 0.01, as indicated. Data are presented as mean ± SD.

The mouse model of ALI was successfully established by intratracheal infusion of LPS. As shown in Fig. 1a, compared with the control group, the LPS group showed pathological changes at 6, 24, and 72 h. With the increased time of LPS stimulation, the lung W/D ratio, BALF protein levels, and BALF cell counts were increased as well (Fig. 1b–d). In conclusion, the severity of LPS-induced ALI was aggravated within 72 h in a time-dependent manner.

The expression levels of FGF21 at different time points were examined in the LPS-induced ALI model. *In vivo*, at the transcriptome and protein levels, the expression of FGF21 in the lung tissue showed a significant increase at 24 and 72 h, but not at 6 h (Fig. 1e and f). As pulmonary epithelial cells form the first physical frontline against external insults, the expression of FGF21 in BEAS-2B cells was detected. At both transcription levels and protein levels in cell culture medium, the expression levels of FGF21 were decreased at 24 and 72 h challenged with LPS (Fig. 1g and h).

FGF21 is a secreted endocrine factor, which is produced predominantly in the liver. Hence, we performed ELISA to examine serum level of FGF21 and RT-PCR to detect the level of FGF21 in the liver. The levels of FGF21 were increased in the serum, which were consistent with the increases in hepatic FGF21 mRNA expression treated with LPS for 24 and 72 h (Fig. 1i and j).

After LPS stimulation, the expression of FGF21 was increased in the lung, serum, and liver while decreased in BEAS-2B cells, which aroused our interest tremendously. Whether FGF21 can be used as a potential marker of injury or may play a compensatory protective role in LPS-induced ALI needs further study. Thus, we investigated the effect upon FGF21 gene deletion as well as upon exogenous administration of FGF21 in mice with LPS-induced ALI.

### The FGF21 Gene Deletion Aggravated Pathological Damage, Inflammatory Infiltration, and Pulmonary Function in LPS-induced Acute Lung Injury Mice Model

**Fig. 2:**
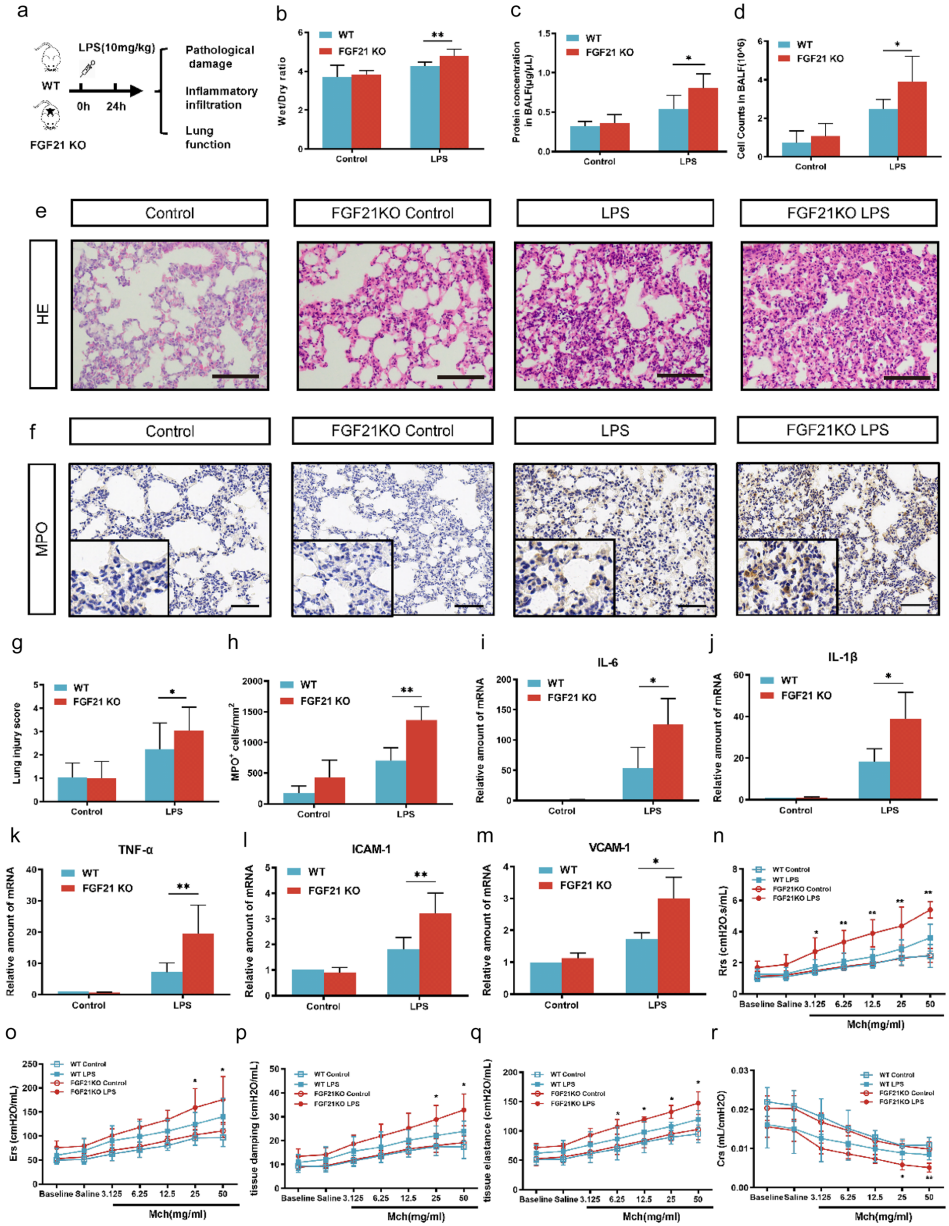
FGF21 deficiency aggravated LPS-induced ALI. **a** The flow-process diagram of establishment of the LPS-induced ALI model in WT and FGF21KO mice. **b** Lung wet/dry ratio of control group, FGF21KO control group, LPS group, and FGF21KO LPS group. After bronchoalveolar lavage fluid (BALF) was collected, the levels of total secretory proteins (*n* = 8) (**c**) and the cells in BALF (*n* = 6) (**d**) of control group, FGF21KO control group, LPS group, and FGF21KO LPS group were assessed. **e** Representative H&E staining image of the lung tissue of control group, FGF21KO control group, LPS group, and FGF21KO LPS group, scale bar = 50 μm (*n* = 20). **f** Immunohistochemical staining of MPO in the lung in control group, FGF21KO control group, LPS group, and FGF21KO group (magnification, × 200 and × 400) (*n* = 15), scale bar = 50 μm. **g** The lung injury score of control group, FGF21KO control group, LPS group, and FGF21KO LPS group was evaluated (*n* = 20). **h** The statistical graph of MPO-positive cells (*n* = 15). **i–m** Real-time PCR results of the expression of IL-6, IL-1β, TNF-α, ICAM-1, and VCAM-1 in control group, FGF21KO control group, LPS group, and FGF21KO LPS group (*n* = 4). The lung function of mice in control group, FGF21KO control group, LPS group, and FGF21KO LPS group was assessed as follows: **n** total lung resistance (Rrs), **o** total lung elastance (Ers), **p** tissue damping (Gtis), **q** tissue elastance (Htis), **r** respiratory system compliance (Crs) (*n* = 4). Multiple comparisons were analyzed by one-way ANOVA. Two-way ANOVA was used in assessment of lung function. *P < 0.05 and **P < 0.01, as indicated. Data are presented as mean ± SD.

To address the influence of FGF21 deficiency on LPS-induced ALI, we assessed the damage of LPS-induced ALI in WT mice and FGF21KO mice in terms of pathological damage, inflammatory infiltration, and pulmonary function (Fig. 2a). As mentioned above, previous studies have already shown that LPS can cause marked pulmonary injury after 24 h (Fig. 1a–d). Therefore, the experiment condition of 24-h time point was selected for subsequent experiments.

To explore the pathological damage caused by LPS-induced ALI following FGF21 deletion, lung tissues and BALF from each group of mice were collected. The results showed that there were obvious increases in the lung W/D ratio, BALF protein levels, and BALF cell counts in FGF21KO LPS–treated mice compared to the mice receiving LPS alone (Fig. 2b–d). In addition, the deletion of FGF21 showed significantly aggravated pathological changes and higher lung injury score under LPS stimulation (Fig. 2e, g). In terms of inflammatory infiltration, the deletion of FGF21 remarkably increased neutrophil infiltration in the lung tissues of LPS-challenged mice, which were evaluated by immunohistochemistry for detecting MPO (Fig. 2f, h). Moreover, the expression levels of inflammatory cytokines including IL-6, IL-1β, TNF-α, ICAM-1, and VCAM-1 were increased in FGF21KO LPS–treated group compared with the LPS-alone group (Fig. 2i–m). Then, the influence of FGF21 deficiency in pulmonary function was investigated. LPS treatment impaired lung function, as observed by its effects after a dose–response methacholine challenge. Compared to the WT LPS group, the FGF21KO LPS group showed more obvious alterations in lung function with significant upregulation of lung resistance (Rrs), lung elastance (Ers), tissue damping, tissue elastance, and downregulation of lung compliance (Crs) (Fig. 2n–r). Besides, there was no sharp distinction between groups of FGF21KO control and WT control in all the above assessment indicators.

In conclusion, these results revealed that FGF21 deletion exacerbated pathological damage, aggravated inflammatory infiltration, and worsened lung function in LPS-induced ALI mice model, which hinted for a possible protective role of FGF21 in LPS-induced ALI.

### Exogenous Administration of FGF21 Alleviated Pathological Damage, Inflammatory Infiltration, and Pulmonary Function in LPS-induced Acute Lung Injury Mice Model

**Fig. 3:**
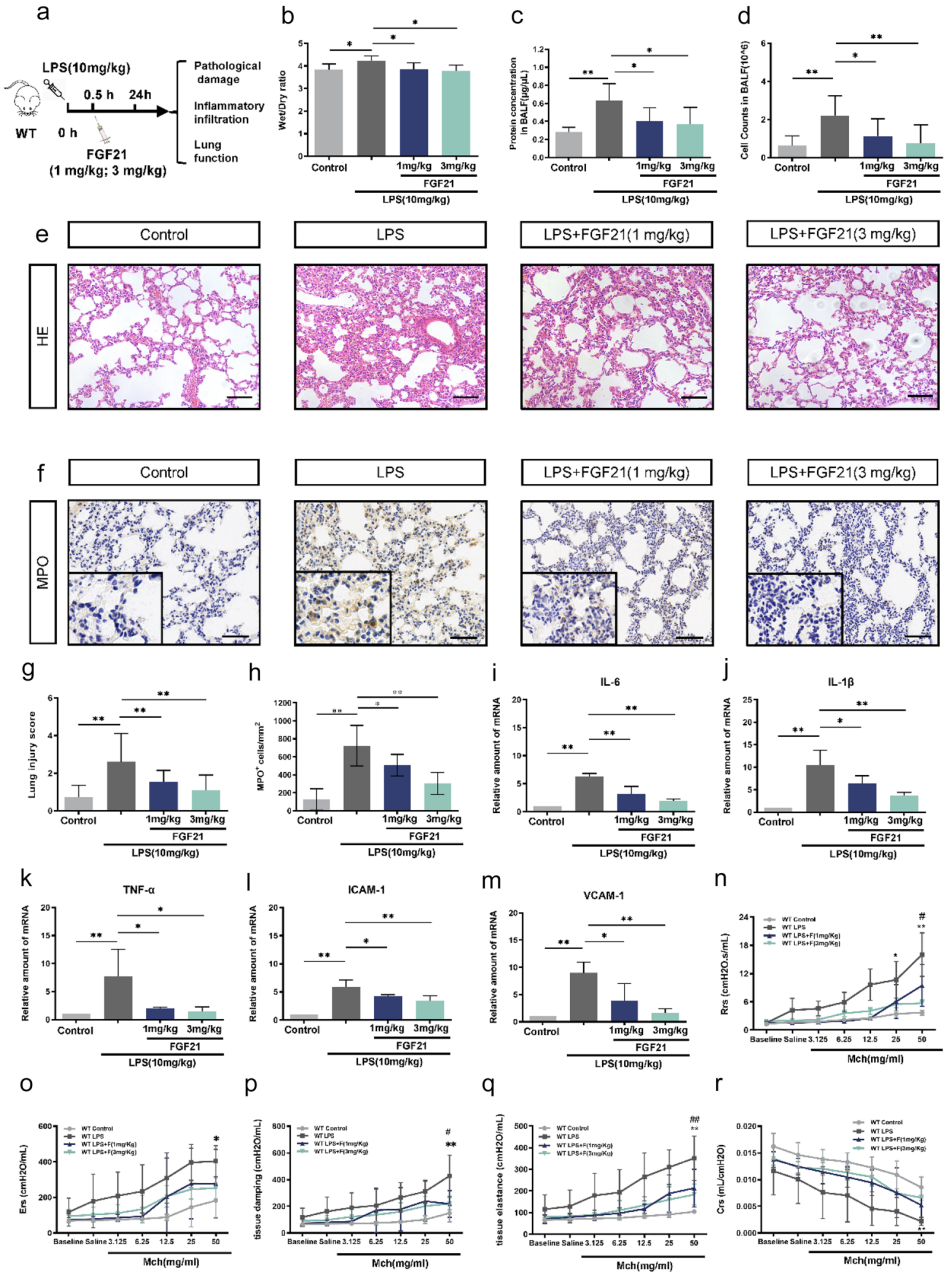
The therapeutic role of FGF21 on the LPS-induced ALI. **a** The flow-process diagram of treatment of FGF21 injection through trachea in LPS-induced ALI mice model. **b** Lung wet/dry ratio of control group, LPS group, LPS + FGF21 (1 mg/kg) group, and LPS + FGF21 (3 mg/kg) group. After BALF was collected, the levels of total secretory proteins (*n* = 5) (**c**) and the cells in BALF (*n* = 6) (**d**) of control group, LPS group, LPS + FGF21 (1 mg/kg) group, and LPS + FGF21 (3 mg/kg) group were assessed. **e** Representative H&E staining image of the lung tissue of control group, LPS group, LPS + FGF21 (1 mg/kg) group, and LPS + FGF21 (3 mg/kg) group (*n* = 20), scale bar = 50 μm. **f** Immunohistochemical staining of MPO revealed neutrophils infiltration in the lung tissue of control group, LPS group, LPS + FGF21 (1 mg/kg) group, and LPS + FGF21 (3 mg/kg) group (magnification, × 200 and × 400) (*n* = 15, scale bar = 50 μm). **g** The lung injury score of control group, LPS group, LPS + FGF21 (1 mg/kg) group, and LPS + FGF21 (3 mg/kg) group was evaluated (*n* = 20). **h** The statistical graph of MPO-positive dots (*n* = 15). **i–m** Real-time PCR results of the expression of IL-6, IL-1β, TNF-α, ICAM-1, and VCAM-1 in control group, LPS group, LPS + FGF21 (1 mg/kg) group, and LPS + FGF21 (3 mg/kg) group (*n* = 4). The lung function of mice in control group, LPS group, LPS + FGF21 (1 mg/kg) group, and LPS + FGF21 (3 mg/kg) group was assessed as follows: **n** total lung resistance (Rrs), **o** total lung elastance (Ers), **p** tissue damping (Gtis), **q** tissue elastance (Htis), **r** respiratory system compliance (Crs) (*n* = 6). Multiple comparisons were analyzed by one-way ANOVA. Two-way ANOVA was used in assessment of lung function. *P < 0.05 and **P < 0.01, as indicated. Data are presented as mean ± SD.

In order to further explore the potential protective effects of FGF21 in LPS-induced ALI, the low (1 mg/kg) and high (3 mg/kg) dose of FGF21 were injected intraperitoneally to mice 0.5 h after intratracheal instillation of LPS (Fig. 3a). Compared with the LPS group, the W/D ratio, protein levels, and cell counts in BALF were remarkably decreased after treatment with FGF21 (Fig. 3b–d). Moreover, exogenous administration of FGF21 after LPS injury significantly reduced alveolar wall thickening, edema, and alveolar collapse and showed lower lung injury score when compared with the LPS group (Fig. 3e, g). In regard to inflammatory infiltration, there was massive recruitment of neutrophils to the lungs under LPS exposure, which was attenuated by FGF21 treatment (Fig. 3f, h). Furthermore, exogenous administration of FGF21 effectively reduced the generation of inflammatory cytokines including IL-6, IL-1β, TNF-α, ICAM-1, and VCAM-1 induced by LPS (Fig. 3i–m). Then, we further investigated whether FGF21 had a protective effect on lung function in LPS-induced ALI. As expected, compared with the LPS group, treatment with FGF21 reduced the upregulation of Rrs, Ers, tissue damping, tissue elastance, and tissue elastance, and lung compliance was ameliorated as well (Fig. 3n–r).

Based on the results above, it was revealed that exogenous administration of FGF21 in mice model of LPS-induced ALI was able to relieve pathological damage, inflammatory infiltration, and lung function. However, there was no significant difference between the 2 different doses of FGF21 we used in this study.

### RNA Sequencing Using LPS-induced ALI Tissues with or Without FGF21 Gene Deletion and Bioinformatics Analysis

The results revealed that FGF21 plays an important role in alleviating LPS-induced ALI. The potential mechanism required further investigation. Therefore, RNA sequencing was performed using lung tissues from 3 WT mice with ALI and 3 FGF21 KO mice with ALI. According to the data matrix, 693 differentially expressed genes (DEGs) were identified, among which 461 genes were upregulated and 232 genes were downregulated (*P* < 0.05, |FC|> 2) (Supplementary Fig. 2A in Supplementary file 1 and Table2 in Supplementary file 2). Subsequently, GO and KEGG pathway enrichment analyses were performed.

**Fig. 4:**
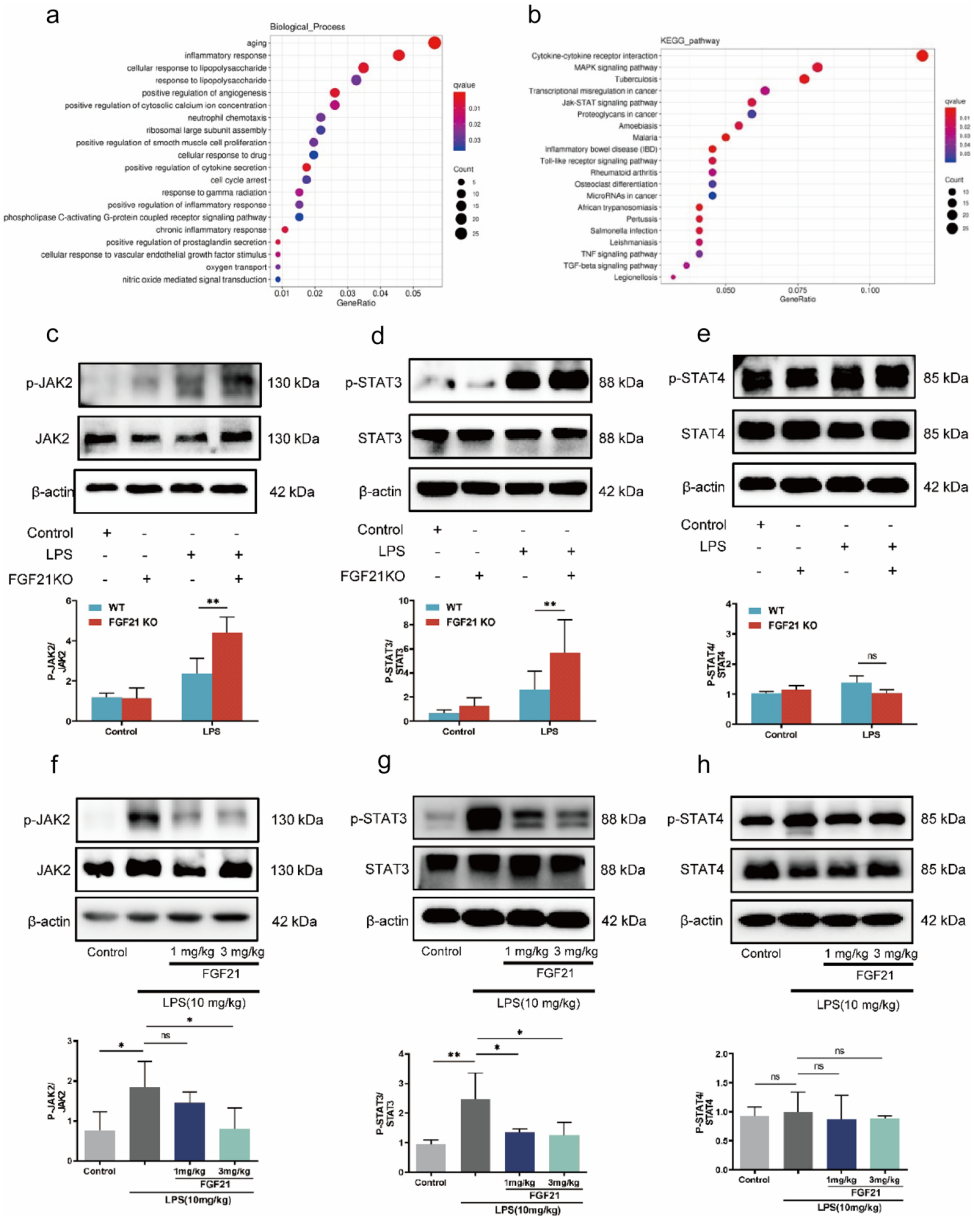
RNA sequencing using LPS-induced ALI tissues with or without FGF21 gene deletion and bioinformatics analysis. **a** Diagrams showed the GO analysis for DEGs representing an association between impacted GO-BP terms and related DEGs. **b** Diagrams showed the KEGG analysis for DEGs to investigate the potential pathways. **c–e** Western blotting results and analysis of p-JAK2/JAK2, p-STAT3/STAT3, and p-STAT4/STAT4 in LPS-induced ALI model of WT mice and FGF21KO mice as well as their controls (*n* = 3). **f–h** Western blotting results and analysis of p-JAK2/JAK2, p-STAT3/STAT3, and p-STAT4/STAT4 in LPS-induced mice treated with or without FGF21 (*n* = 3). Multiple comparisons were analyzed by one-way ANOVA. *P < 0.05 and **P < 0.01, as indicated. Data are presented as mean ± SD.

GO enrichment analysis of DEGs in the molecular function (MF) category revealed that these genes were mainly enriched in the calcium ion binding, cytokine activity, and rRNA binding (Supplementary Fig. 2B in Supplementary file 1). In the cellular component (CC) domain, the top 3 categories were “extracellular space,” “cell surface,” and “neuronal cell body” (Supplementary Fig. 2C in Supplementary file 1). In terms of biological process (BP), the results showed that aging, inflammatory response, and cellular response to LPS were ranked in the top 3 (Fig. 4a). Then, KEGG analysis revealed that the DEGs were mainly enriched in the cytokine-cytokine receptor response, MAPK signaling pathway, and JAK2/STAT3 signaling pathway (Fig. 4b). Considering the significant correlation between cytokine-cytokine receptor interaction and the JAK2/STAT3 signaling pathway, we hypothesized that FGF21 might reduce LPS-induced ALI by regulating the JAK2/STAT3 signaling pathway. To verify this hypothesis, western blotting was performed to detect the effect of FGF21 on the JAK2/STAT3 signaling pathway upon LPS treatment. *In vivo*, the levels of p-JAK2 and p-STAT3 in the LPS group were higher than those in the control group. These changes were aggravated upon FGF21 deletion, while no apparent changes in the phosphorylation levels of STAT4 were noted between the LPS group and FGF21KO LPS group (Fig. 4c–e). In contrast, FGF21 treatment clearly reduced LPS-induced increase in p-JAK2 and p-STAT3 levels (Fig. 4f and g). However, it did not affect the phosphorylation levels of STAT4 (Fig. 4h).

Taken together, through GO enrichment analysis, it was suggested that the deletion of FGF21 might aggravate the inflammation and increase responsiveness to LPS, which was in accordance with the *in vivo* experiment. Furthermore, combining the results of KEGG enrichment analysis and western blotting, it was found that FGF21 might alleviate LPS-induced ALI via JAK2/STAT3 signaling pathway.

### Coumermycin A1 Inhibits the Therapeutic Effect of FGF21 on LPS-induced ALI Mice

**Fig. 5:**
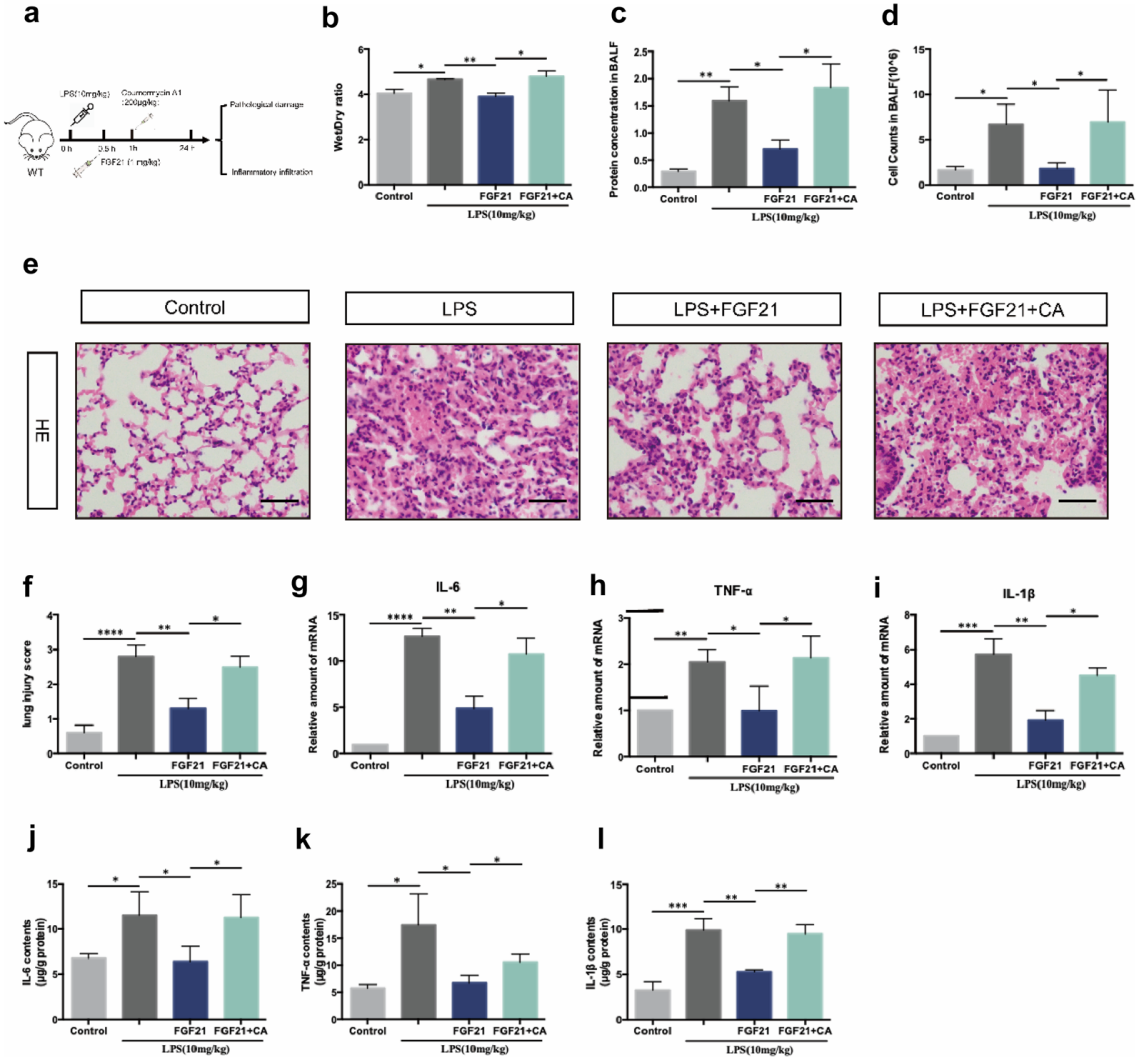
JAK2 activator inhibits the therapeutic effect of FGF21 on LPS-induced ALI mice. **a** The flow-process diagram of treatment of FGF21 injection through trachea in LPS-induced ALI mice model. **b** Lung wet/dry ratio of control group, LPS group, LPS + FGF21 group, and LPS + FGF21 + CA group. After BALF was collected, the levels of total secretory proteins (*n* = 4) (**c**) and the cells in BALF (*n* = 4) (**d**) of control group, LPS group, LPS + FGF21 group, and LPS + FGF21 + CA group were assessed. **e** Representative H&E staining image of the lung tissue of control group, LPS group, LPS + FGF21 group, and LPS + FGF21 + CA group (*n* = 10), scale bar = 50 μm. **f** The lung injury score of control group, LPS group, LPS + FGF21 group, and LPS + FGF21 + CA group was evaluated (*n* = 10). **g–i** The mRNA level of IL-6, IL-1β, and TNF-α of lung tissue in control group, LPS group, LPS + FGF21 group, and LPS + FGF21 + CA group (*n* = 4). **j–l** The protein level of IL-6, IL-1β, and TNF-α in BALF in control group, LPS group, LPS + FGF21 group, and LPS + FGF21 + CA group (*n* = 4). Multiple comparisons were analyzed by one-way ANOVA. *P < 0.05 and **P < 0.01, as indicated. Data are presented as mean ± SD.

To further investigate the relationship between FGF21 and JAK2/STAT3 signaling pathway in ALI progression, Coumermycin A1 (CA), a classic agonist of JAK2, was used to give to LPS-induced ALI mice after treated with FGF21 (3 mg/kg) for 0.5 h (Fig. 5a). The wet/dry ratio, cell counts, protein levels in BALF, and pathological damage of lung tissue in LPS + FGF21 group were alleviated compared with LPS group, while the therapeutic effect of FGF21 was reversed by CA (Fig. 5b–f). In the meantime, the mRNA levels as well as protein levels of cytokines in lung tissue such as IL-6, IL-1β, and TNF-α were decreased after treatment of FGF21 and risen again when treated with additional CA (Fig. 5g–l).

In short, these results proved that FGF21 relieves LPS-induced ALI through suppressing JAK2/STAT3 signaling pathway.

### FGF21 Treatment Suppresses Activation of the JAK2/STAT3 Pathway and p-STAT3 Nuclear Translocation in BEAS-2B Cells

To further explore the effect of FGF21 on the JAK2/STAT3 signaling pathway, siRNA was used to knock down the expression of FGF21 in BEAS-2B cells and the most effective siRNA (si-FGF21-3) was selected for subsequent experiments (Supplementary Fig. 3A in Supplementary file 1). In addition, the expression levels of STAT3 and FGF21 were increased using overexpression plasmids and the efficiency of the plasmids was confirmed (Supplementary Fig. 3B, C in Supplementary file 1).

**Fig. 6:**
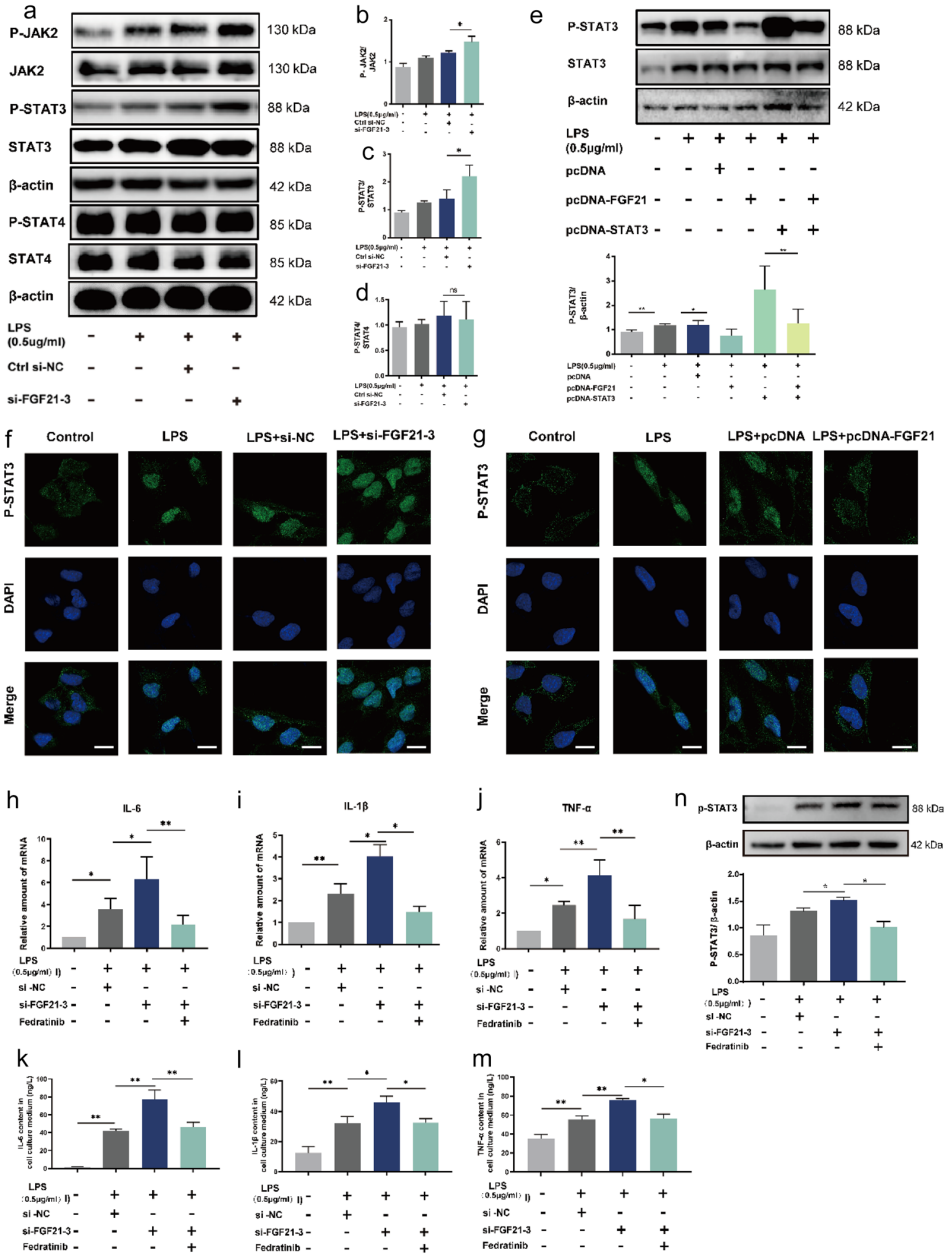
FGF21 treatment suppresses activation of the JAK2/STAT3 pathway and p-STAT3 nuclear translocation in BEAS-2B cells. **a–d** Western blotting results and analysis of p-JAK2/JAK2, p-STAT3/STAT3, and p-STAT4/STAT4 after the silence of FGF21 in the indicated cell models (*n* = 3). **e** Western blotting results and analysis of p-STAT3/STAT3 after simultaneous overexpression of FGF21 and STAT3 in the indicated cell models (n = 3). **f** and **g** Representative images of immunofluorescence analysis of p-STAT3 in the indicated cell models (scale bar = 20 μm). **h–j** Relative amount of IL-6, IL-1β, and TNF-α mRNA of BEAS-2B in control group, LPS + si-NC group, LPS + si-FGF21-3 group, and LPS + si-FGF21-3 + Fedratinib group (*n* = 4). **k–m** The protein level of IL-6, IL-1β, and TNF-α in culture medium of BEAS-2B cells in control group, LPS + si-NC group, LPS + si-FGF21-3 group, and LPS + si-FGF21-3 + Fedratinib (*n* = 3). **n** Western blotting results and analysis in control group, LPS + si-NC group, LPS + si-FGF21-3 group, and LPS + si-FGF21-3 + Fedratinib group (*n* = 3). Multiple comparisons were analyzed by one-way ANOVA. *P < 0.05 and **P < 0.01, as indicated. Data are presented as mean ± SD.

As expected, the results showed that the p-JAK2 and p-STAT3 protein levels were significantly elevated in the LPS + si-FGF21-3 group compared with the LPS group, while there was no statistically significant difference in p-STAT4 protein levels between the 2 groups (Fig. 6a–d). Moreover, western blotting revealed that the overexpression of FGF21 effectively reduced the level of p-STAT3 following LPS stimulation. Next, it was demonstrated that the expression of p-STAT3 was increased in cells transfected with the STAT3 plasmid after LPS treatment and this increase was reversed by the simultaneous transfection of FGF21 plasmid (Fig. 6e).

Then, immunofluorescence analysis was performed to detect the distribution of p-STAT3. As shown in Fig. 6f, it was revealed that LPS significantly enhanced the nuclear translocation of p-STAT3 in BEAS-2B cells, whereas siRNA-mediated knockdown of FGF21 further increased its translocation into the nucleus. However, overexpression of FGF21 remarkably reduced the nuclear translocation of p-STAT3 compared to the LPS group (Fig. 6g). These findings indicated that FGF21 could decrease the translocation of p-STAT3 into the nucleus in pulmonary epithelial cells upon exposure to LPS.

Furthermore, we detected the influence of FGF21 on LPS-induced ALI after inhibiting JAK/STAT3 signal pathway by Fedratinib (a JAK2 selective inhibitor) *in vitro*. CCK8 assay was utilized to evaluate cytotoxicity and to determine the non-toxic drug concentration of Fedratinib (Supplementary Fig. 3D in Supplementary file 1). The results showed that siRNA-mediated knockdown of FGF21 has further increased the expression levels of IL-6, IL-1β, and TNF-α after LPS treatment in transcription level. However, the aggravation of inflammatory reaction by silencing the FGF21 gene was alleviated by Fedratinib (Fig. 6h–j). Similarly, as shown in Fig. 6k–m, compared with LPS + si-FGF21-3 group, the expression of IL-6, IL-1β, and TNF-α in protein levels was downregulated in LPS + si-FGF21-3 + Fedratinib group. In addition, the expression level of p-STAT3 was increased in LPS + si-FGF21-3 group compared with LPS + si-NC group and the upregulation was blocked by treatment with Fedratinib (Fig. 6n). Overall, the results revealed that FGF21 might alleviate LPS-induced ALI through inhibiting JAK2/STAT3 signaling pathway.

## DISCUSSION

ALI/ARDS is a major source of morbidity and mortality of patients in intensive care units (ICUs). Owing to their diverse etiologies and numerous pathogenic links, in-depth exploration of possible treatment options for ALI/ARDS has been the subject of great interest in medical research.

FGF21 is a critical regulator of lipid and glucose metabolism [30, 31]. Previous studies have focused on the pharmacological effects and clinical potential of FGF21 in cardiovascular diseases and metabolic diseases such as diabetes mellitus, obesity, and insulin resistance [8, 32–35]. Several reports have found that serum levels of FGF21 were increased in subjects with non-alcoholic fatty liver disease, hyperlipidemia, hypertension, and atherosclerosis [36–39]. The circulating levels of FGF21 were also found to be higher in patients with sepsis and systemic inflammatory response syndrome (SIRS) than in healthy controls [12]. However, the expression level of FGF21 in LPS-induced ALI has not yet been detected.

In this study, the expression of FGF21 was significantly increased in lung tissues from mice with LPS-induced ALI compared to that in healthy controls after 24 h, while the expression of FGF21 in BEAS-2B cells was consistently decreased upon LPS exposure. The different expression trend of FGF21 in the lung and BEAS-2B cells indicated that there might be an additional supply of FGF21 to the lungs during ALI. FGF21 exerts a wide range of tissue-specific autocrine, paracrine, and endocrine metabolic effects [40, 41]. Subsequent experiments revealed that the levels of FGF21 were increased in both the serum and liver response to LPS. The elevated levels of FGF21 in the lung tissues may be due to elevated circulating levels of FGF21. The increased levels of serum FGF21 might be mainly due to enhanced expression of FGF21 in the liver and its subsequent secretion into the circulation. Our results suggested a compensatory FGF21 overproduction in response to LPS-induced ALI. Due to limited experimental conditions, the study presented some deficiencies. Further researches require the construction of liver-specific FGF21 knockout mice and lung-specific FGF21 knockout mice to explore origin of organ that secreted FGF21 under LPS stimulation. In addition, except for the endocrine role of FGF21, FGF21 also acts in a paracrine and autocrine way. However, the results of this study indicated that the expression of FGF21 in BEAS-2B cells consistently decreased upon LPS exposure [42]. Whether other types of cells in the lung tissue express FGF21 to function in a paracrine manner after LPS stimulation requires further investigation.

We further examined whether FGF21 exerts a protective compensatory response or is merely an injury-related marker for ALI. Several recent studies have suggested that FGF21 played an important role in the regulation of inflammation [43–45]. Jing Gao *et al*. found that FGF21 suppressed both apoptosis and inflammation via the TLR4/MYD88/NF-κB signaling pathway in LPS-induced ALI [46]. It has also been reported that FGF21 ameliorated the dysfunction and inflammatory response in HPMECs through SIRT1-mediated NF-κB deacetylation [14]. However, these findings require further investigation. In our study, it was proved that deletion of FGF21 gene in mice model of LPS-induced ALI enhanced tissue injury, facilitated the production of inflammatory mediators, and accelerated impaired pulmonary mechanics. In addition, GO enrichment analysis indicated that deletion of FGF21 gene might aggravate the inflammatory response in ALI, which was consistent with the above results. Exogenous administration of FGF21 reduced the above effects. Thus, it was validated that FGF21 acts as a protective factor against LPS-induced ALI.

To further elucidate the underlying molecular mechanism, RNA sequencing and bioinformatics analysis were performed using tissues from WT mice with LPS-induced ALI and FGF21 KO mice with LPS-induced ALI. Jing Gao *et al*. found that FGF21 suppressed LPS-induced ALI via the TLR4/MYD88/NF-κB signaling pathway [46]. However, KEGG analysis showed that cytokine-cytokine receptor response, MAPK, and JAK-STAT signaling pathways were probably involved in the alleviation of LPS-induced ALI mediated by FGF21. The cytokine-cytokine receptor interaction pathway comprises chemokines as well as several cytokines and growth factors, including IL-6 [17]. In classical signaling, the binding of IL-6 to the IL-6 receptor, which interacts with the membrane protein gp130, canonically initiates the JAK2/STAT3 signaling pathway and subsequently exerts pleiotropic effects on the immune system [47]. It has also been reported that ulinastatin protected rats from sepsis-induced ALI by suppressing the JAK/STAT3 signaling pathway [48]. Moreover, miR-216a attenuates LPS-induced inflammatory injury by inhibiting the JAK2/STAT3 and NF-κB signaling pathways [49]. The above studies indicated that the JAK2/STAT3 signaling pathway is widely involved in inflammation, which is also activated in LPS-induced ALI. However, to our knowledge, no relevant previous studies have reported that the JAK2/STAT3 signaling pathway might be involved in FGF21-medaited alleviation of LPS-induced ALI.

We found that the expression levels of p-JAK2 and p-STAT3 in the lung tissues of FGF21 KO mice upon LPS treatment were significantly increased, while exogenous administration of FGF21 reversed these levels. Moreover, JAK2 activator hindered the therapeutic effect of FGF21 in LPS-induced mice and cell experiments showed a markedly aggravated inflammatory phenotype of FGF21 silencing, which can be reversed by JAK2 inhibitor. STAT3 phosphorylation and nuclear translocation were increased in BEAS-2B cells upon FGF21 silencing and decreased in BEAS-2B cells upon forced overexpression of FGF21. Thus, based on literature and bioinformatics analysis, as well as *in vitro* and *in vivo* experimental results, FGF21 acts as a protective factor and possibly alleviates LPS-induced ALI by inhibiting the JAK2/STAT3 signaling pathway. However, whether FGF21 inhibits the JAK2/STAT3 signaling pathway through direct or indirect effects requires further investigation. In addition, bioinformatics analysis suggested that MAPK signaling pathway could be involved in FGF21 alleviating LPS-induced ALI. The MAPK pathway is one of the common intersection pathways associated with cell proliferation, stress, inflammation, differentiation, functional synchronization, transformation, apoptosis, and other signal transduction pathways [50–52]. In our future studies, we seek to further clarify whether the MAPK signaling pathway is involved in FGF21-mediated repair of the air-blood barrier.

## CONCLUSION

**Fig. 7:**
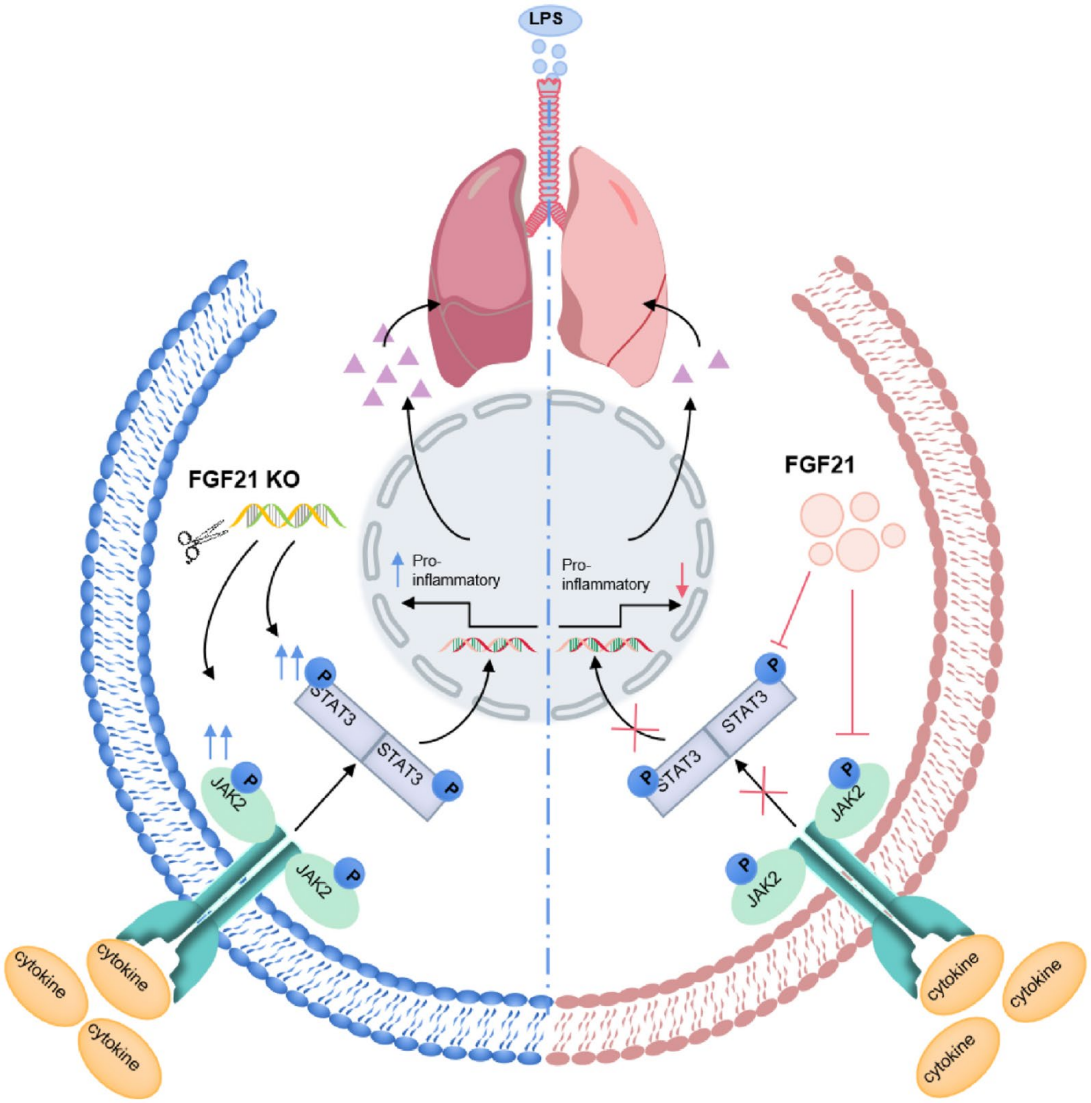
The mechanisms of FGF21 protect against ALI through suppressing JAK2/STAT3 signaling pathway.

In conclusion, FGF21 expression levels were enhanced in lung tissues, serum, and liver tissues of mice with LPS-induced ALI, but decreased in BEAS-2B cells. The deficiency of FGF21 aggravated LPS-induced ALI in mice, and exogenous administration of FGF21 reduced the effect. In addition, the protective effect of FGF21 might possibly be due to inhibition of the JAK2/STAT3 signaling pathway (Fig. 7). These findings might provide new targets for curing ALI/ARDS in the future.

### Supplementary Information

Below is the link to the electronic supplementary material.

Supplementary file1 (XLSX 38 KB)

Supplementary file2 (PDF 1910 KB)

## Data Availability

The raw data presented in this study will be made available by the authors, without undue reservation.
