# Supplementary material for: Fibroblast Growth Factor 21 Relieves Lipopolysaccharide-Induced Acute Lung Injury by Suppressing JAK2/STAT3 Signaling Pathway
Source: Inflammation. 2023 Oct 21;47(1):209–26. doi: 10.1007/s10753-023-01905-3 (PMC10799097; doi:10.1007/s10753-023-01905-3)
Supplement: Supplementary file 2 — Supplementary file2 (PDF 1910 KB) [file 10753_2023_1905_MOESM2_ESM.pdf]

Table1 Primer sequences used for quantitative real-time polymerase chain reaction(RT-PCR)

Gene name	
human FGF21	Forward: 5'-ATGGATCGCTCCACTTTGACC-3'
	Reverse: 5'-GGGCTTCGGACTGGTAAACAT-3'
mouse FGF21	Forward: 5'-GATGACGACCAAGACACTGAAGCC-3'
	Reverse: 5'-CTCCAGCAGCAGTTCTCTGAAGC-3'
mouse IL-6	Forward:5'-GAGGATACCACTCCCAACAGACC-3'
	Reverse: 5'-AAGTGCATCATCGTTGTTCATACA-3'
mouse IL-1 β	Forward: 5'-ACTCCTTAGTCCTCGGCCA-3'
	Reverse: 5'-CCATCAGAGGCAAGGAGGAA-3'
mouse TNF-α	Forward: 5'-TGATCCGCGACGTGGAA-3'
	Reverse: 5'-ACCGCCTGGAGTTCTGGAA-3'
mouse ICAM-1	Forward: 5'-GCCTTGGTAGAGGTGACTGAG-3'
	Reverse: 5'-AAGTGCATCATCGTTGTTCATACA-3'
mouse VCAM-1	Forward: 5'-TGCCGAGCTAAATTACACATTG-3'
	Reverse: 5'-CCTTGTGGAGGGATGTACAGA-3'
mouse β-actin	Forward: 5'-CCGTGAAAAGATGACCCAGA-3'
	Reverse: 5'-TACGACCAGAGGCATACAG-3'

FGF21: fibroblast growth factor 21; IL-6, interleukin-6; IL-1β, interleukin-1β; TNF-α, tumor necrosis factor-α; ICAM-1, intercellular cell adhesion molecule-1; VCAM-1, vascular cell adhesion molecule-1;

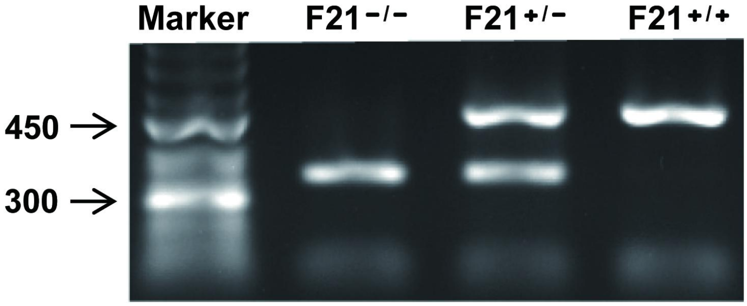

Supplementary Fig.1 FGF21 knock out genetic identification: agarose gel electropherogram of FGF21 in mice

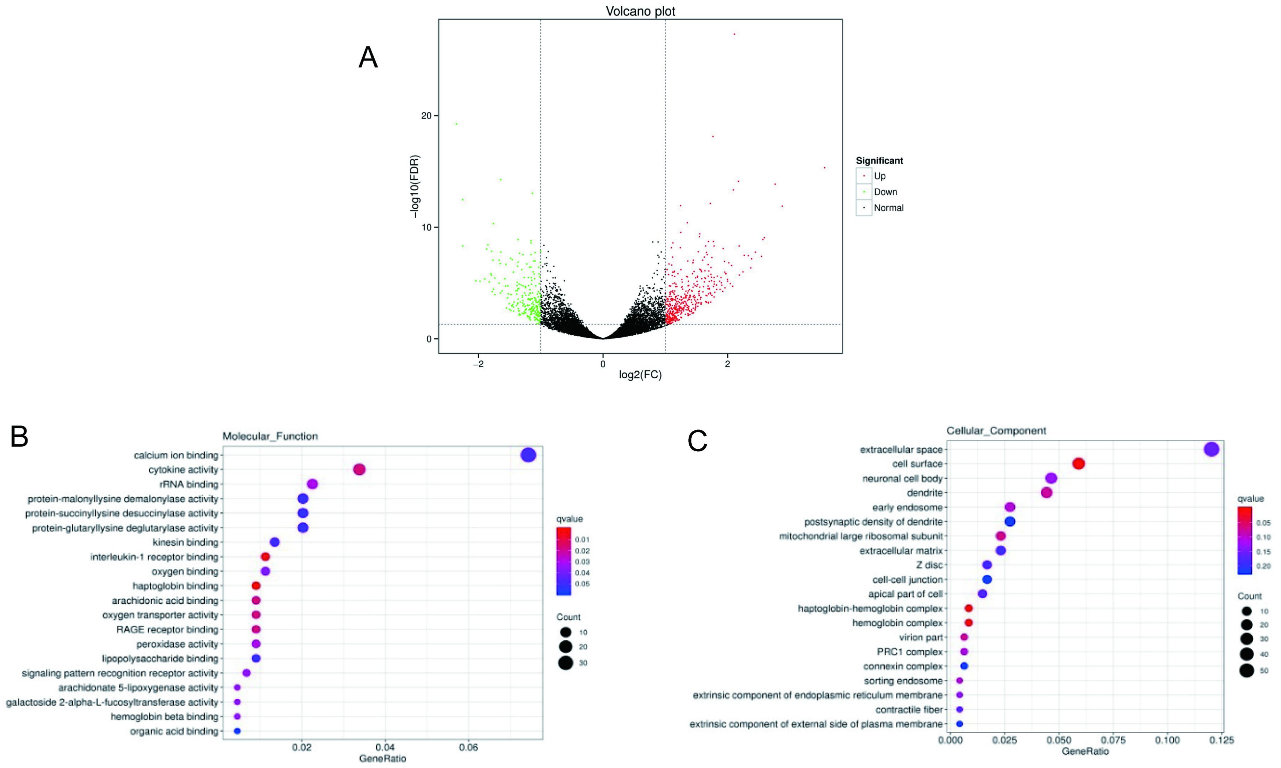

Supplementary Fig. 2 RNA sequencing and bioinformatics analysis (A) The volcano plot of molecular function. GO enrichment analysis of molecular function (B) and cellular component (C).

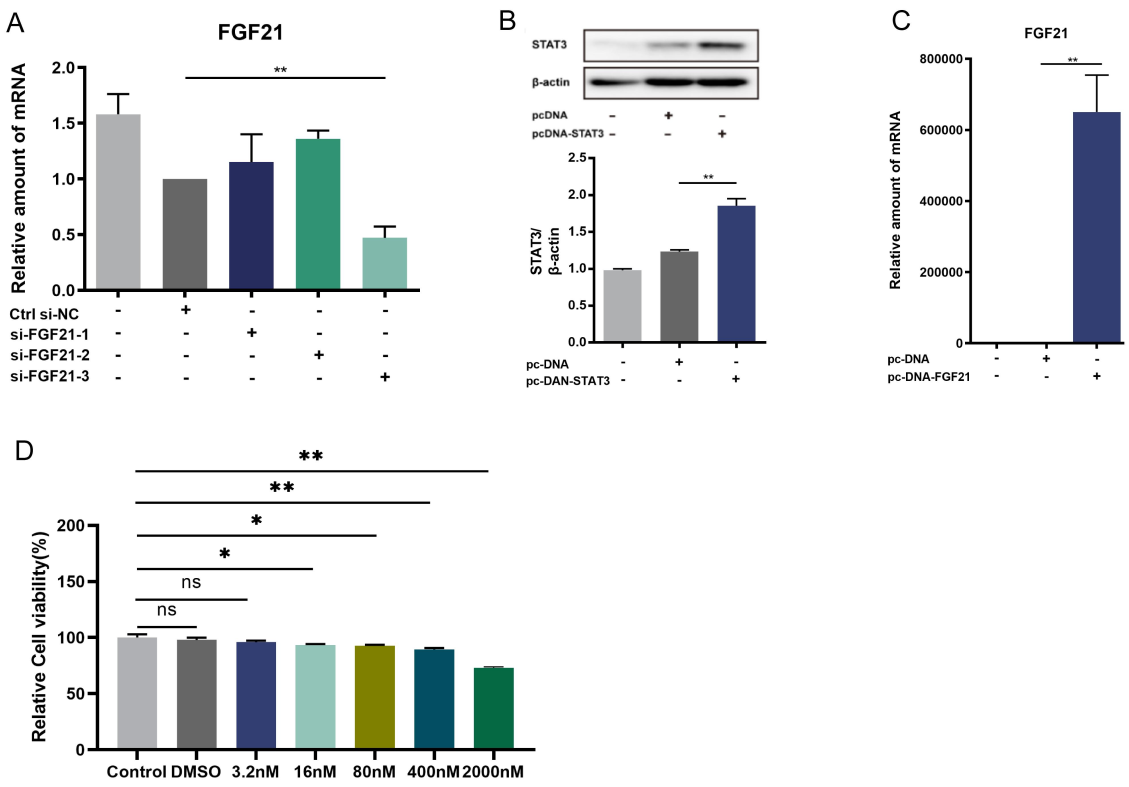

Supplementary Fig. 3 Validation of Si-RNA overexpression plasma and CCK-8 test of Fedratinib (A) Validation of FGF21 silencing efficiency. (B) Verification of overexpression efficiency of STAT3. (C) Verification of overexpression efficiency of FGF21. (D) The CCK-8 test of Fedratinib on BEAS-2B Cells (n=3). Multiple comparisons were analyzed by one-way ANOVA. *P < 0.01 and **P < 0.05.
